# Neuropsychological outcome after cardiac arrest: results from a sub-study of the targeted hypothermia versus targeted normothermia after out-of-hospital cardiac arrest (TTM2) trial

**DOI:** 10.1186/s13054-023-04617-0

**Published:** 2023-08-26

**Authors:** Erik Blennow Nordström, Susanna Vestberg, Lars Evald, Marco Mion, Magnus Segerström, Susann Ullén, John Bro-Jeppesen, Hans Friberg, Katarina Heimburg, Anders M. Grejs, Thomas R. Keeble, Hans Kirkegaard, Hanna Ljung, Sofia Rose, Matthew P. Wise, Christian Rylander, Johan Undén, Niklas Nielsen, Tobias Cronberg, Gisela Lilja

**Affiliations:** 1 Neurology, Department of Clinical Sciences Lund, Skane University Hospital, Lund University, Lund, Sweden; 2 Department of Psychology, Lund University, Lund, Sweden; 3 Hammel Neurorehabilitation Centre and University Research Clinic, Hammel, Denmark; 4 Essex Cardiothoracic Centre, Mid and South Essex NHS Foundation Trust, Basildon, UK; 5 Medical Technology Research Centre, Anglia Ruskin School of Medicine, Chelmsford, UK; 6 Department of Neurology and Department of Cardiology, Sahlgrenska University Hospital, Gothenburg, Sweden; 7 Clinical Studies Sweden – Forum South, Skane University Hospital, Lund, Sweden; 8 Department of Cardiology, Aarhus University Hospital, Aarhus, Denmark; 9 Intensive and Perioperative Care, Department of Clinical Sciences Lund, Skane University Hospital, Lund University, Malmö, Sweden; 10 Department of Intensive Care Medicine and Department of Clinical Medicine, Aarhus University Hospital and Aarhus University, Aarhus, Denmark; 11 Research Centre for Emergency Medicine, Emergency Department and Department of Clinical Medicine, Aarhus University Hospital and Aarhus University, Aarhus, Denmark; 12 Clinical Psychology, Cardiff and Vale University Health Board, NHS Wales, Cardiff, UK; 13 Adult Critical Care, University Hospital of Wales, Cardiff, UK; 14 Anaesthesiology and Intensive Care Medicine, Department of Surgical Sciences, Uppsala University, Uppsala, Sweden; 15 Operation and Intensive Care, Hallands Hospital Halmstad, Halmstad, Sweden; 16 Anesthesiology and Intensive Care, Department of Clinical Sciences Lund, Helsingborg Hospital, Lund University, Lund, Sweden

**Keywords:** Hypoxic-ischemic encephalopathy, Heart arrest, Myocardial infarction, Cognitive impairment, Cardiovascular disease, Outcome

## Abstract

**Background:**

Cognitive impairment is common following out-of-hospital cardiac arrest (OHCA), but the nature of the impairment is poorly understood. Our objective was to describe cognitive impairment in OHCA survivors, with the hypothesis that OHCA survivors would perform significantly worse on neuropsychological tests of cognition than controls with acute myocardial infarction (MI). Another aim was to investigate the relationship between cognitive performance and the associated factors of emotional problems, fatigue, insomnia, and cardiovascular risk factors following OHCA.

**Methods:**

This was a prospective case–control sub-study of The Targeted Hypothermia versus Targeted Normothermia after Out-of-Hospital Cardiac Arrest (TTM2) trial. Eight of 61 TTM2-sites in Sweden, Denmark, and the United Kingdom included adults with OHCA of presumed cardiac or unknown cause. A matched non-arrest control group with acute MI was recruited. At approximately 7 months post-event, we administered an extensive neuropsychological test battery and questionnaires on anxiety, depression, fatigue, and insomnia, and collected information on the cardiovascular risk factors hypertension and diabetes.

**Results:**

Of 184 eligible OHCA survivors, 108 were included, with 92 MI controls enrolled. Amongst OHCA survivors, 29% performed *z*-score ≤ − 1 (at least borderline–mild impairment) in ≥ 2 cognitive domains, 14% performed *z*-score ≤ − 2 (major impairment) in ≥ 1 cognitive domain while 54% performed without impairment in any domain. Impairment was most pronounced in episodic memory, executive functions, and processing speed. OHCA survivors performed significantly worse than MI controls in episodic memory (mean difference, MD = − 0.37, 95% confidence intervals [− 0.61, − 0.12]), verbal (MD = − 0.34 [− 0.62, − 0.07]), and visual/constructive functions (MD = − 0.26 [− 0.47, − 0.04]) on linear regressions adjusted for educational attainment and sex. When additionally adjusting for anxiety, depression, fatigue, insomnia, hypertension, and diabetes, executive functions (MD = − 0.44 [− 0.82, − 0.06]) were also worse following OHCA. Diabetes, symptoms of anxiety, depression, and fatigue were significantly associated with worse cognitive performance.

**Conclusions:**

In our study population, cognitive impairment was generally mild following OHCA. OHCA survivors performed worse than MI controls in 3 of 6 domains. These results support current guidelines that a post-OHCA follow-up service should screen for cognitive impairment, emotional problems, and fatigue.

***Trial registration*:**

ClinicalTrials.gov, NCT03543371. Registered 1 June 2018.

**Supplementary Information:**

The online version contains supplementary material available at 10.1186/s13054-023-04617-0.

## Background

The survival rate for adult out-of-hospital cardiac arrest (OHCA) is poor but has increased over the recent decades, with an estimated global 1-year survival rate of 7.7% [1]. This is associated with a growing need for follow-up of both the seen and unseen consequences after OHCA [2]. Circulatory standstill and the subsequent low-flow state that characterizes cardiac arrest results in an immediate interruption of oxygen supply and may lead to hypoxic-ischemic brain injury [3]. Cognitive impairments, especially in the cognitive domains of memory, attention/processing speed, and executive functioning, are reported in about half of survivors [4, 5]. However, these domains are the most thoroughly investigated and smaller studies indicate that verbal and visual/constructive functions could be affected too [6, 7]. Even mild cognitive impairment after OHCA can lead to reduced societal participation and major cognitive impairment has a substantial effect on daily function [8, 9]. An earlier study reported that cognitive impairment was common six months after OHCA but found only minor cognitive differences between OHCA survivors and a risk factor matched control group with acute ST-elevation myocardial infarction (STEMI) [10]. This earlier study mainly used screening tests and focused on a limited number of cognitive domains. Furthermore, OHCA is a risk factor for the development of emotional problems [11]. Brain injury and associated factors such as emotional problems, fatigue, and cardiovascular risk factors might also contribute to cognitive impairment post-cardiac arrest. However, the nature of cognitive impairment and the role of associated factors should be further investigated with more sensitive tests and comprehensive data.

Our aims in this study, using detailed neuropsychological tests of cognition, were to: provide detailed information on cognitive impairment in OHCA survivors at 7 months, compare the cognitive performance to a matched cohort of participants following acute myocardial infarction (MI) without cardiac arrest (and therefore without the risk of hypoxic-ischemic brain injury), and investigate the relationship between cognitive performance and the associated factors of emotional problems, fatigue, insomnia, and cardiovascular risk factors following OHCA. We hypothesized that OHCA survivors would perform significantly worse on the neuropsychological tests compared to MI controls.

## Methods

### Study design

This predefined case–control sub-study is a part of the international, multicenter Targeted Hypothermia versus Targeted Normothermia after Out-of-Hospital Cardiac Arrest (TTM2) trial [12, 13], in which 8 of 61 TTM2-sites participated. The study protocol has been published [14] and the study registered at ClinicalTrials.gov, NCT03543371.

### Participants and procedure

In the TTM2-trial, comatose adult survivors of OHCA with a presumed cardiac or unknown cause were randomized to targeted hypothermia at 33 °C or targeted normothermia with early treatment of fever (core temperature ≤ 37.8 °C) [12, 13]. At the six-month TTM2-trial follow-up meeting [15], survivors at the selected sites in Sweden, Denmark, and the United Kingdom were invited to this sub-study that was performed as a separate face-to-face meeting. One site in each country was responsible for recruiting a non-arrest control group of participants with acute MI (STEMI and non-STEMI) who had undergone coronary angiography and appropriate revascularization. As earlier described [14], the MI controls were matched to date of cardiac event (± 4 weeks), sex, and age (best match) at an intended 1:1 ratio, and participated in a similar visit. Between July 2018 and January 2021, the sub-study was performed approximately 7 months post-cardiac event up to 12 months at the latest. In comparison to the TTM2-trial, additional exclusion criteria for this sub-study were age ≥ 80, a clinical diagnosis of major neurocognitive disorder (dementia) before the cardiac event, inability to speak the local language well enough to complete the assessment without assistance from an interpreter, inability to meet for a face-to-face examination, active drug abuse, and Clinical Frailty Scale Index ≥ 8, indicating very severe frailty [14, 16]. The TTM2-trial and this sub-study received approval by ethics committees in all participating countries. All included participants gave their written and oral informed consent.

### Outcome assessments

*Neuropsychological test battery* Six cognitive domains were assessed, italicized below, based on the subsequent neuropsychological tests.

- *Verbal* Wechsler Adult Intelligence Scale—Fourth Edition (WAIS-IV) Vocabulary [17], Delis-Kaplan Executive Function System (D-KEFS) Verbal Fluency [18].
- *Visual/constructive* WAIS-IV Block Design, Matrix Reasoning [17].
- *Working memory* WAIS-IV Digit Span [17], Wechsler Memory Scale – Third Edition (WMS-III) Spatial Span [19].
- *Episodic memory* Rey Auditory Verbal Learning Test (RAVLT) [20], WMS-III Logical Memory [19], Brief Visuospatial Memory Test-Revised (BVMT-R) [21].
- *Processing speed* Trail Making Test (TMT) A [22], D-KEFS Color-Word Interference Test (CWIT) 1 & 2 [18].
- *Executive functions* TMT B [22], D-KEFS CWIT 3 [18].

Details on the neuropsychological tests are presented in the study protocol [14]. The raw scores were converted to *z*-scores according to age and, when available, education-based population norms. Negative *z*-scores reflect worse scores than the population mean. The *z*-scores of individual neuropsychological tests were combined to the 6 cognitive domains. For each domain a neuropsychological composite score was computed (*z*-scores with normative *M* = 0; *SD* = 1). Scores ≤ − 1 *SD*, equal to a *z*-score of ≤ − 1, are generally considered indicative of possible cognitive impairment [23]. Using classifications for mild and major neurocognitive disorder from the Diagnostic and Statistical Manual of Mental Disorders—5th Edition [23], we described *z*-scores ≤ − 1 as at least borderline–mild cognitive impairment, ≤ − 2 as major impairment, while *z*-scores > − 1 were considered without impairment.

*Questionnaires on emotional problems, fatigue, and insomnia* Before or after the visit, participants filled out questionnaires on self-reported symptoms within the subsequent areas. *Emotional problems* Hospital Anxiety and Depression Scale (HADS) with the anxiety (HADS-A) and depression (HADS-D) subscales [24]; *fatigue* Multidimensional Fatigue Inventory (MFI-20) with the five dimensions General fatigue, Physical fatigue, Reduced activity, Reduced motivation, and Mental fatigue [25]; *insomnia* Minimal Insomnia Symptom Scale (MISS) [26]. When available, cut-scores were used to describe possible clinical conditions (HADS-A and HADS-D: ≥ 8; MISS: ≥ 6) [24, 26]. Higher scores reflect more reported symptoms on all questionnaires.

*Functional outcome* Modified Rankin Scale (mRS) was the clinician-reported functional outcome measure assessed during the six-month TTM2-trial follow-up [27]. The scale ranges from 0 (no symptoms) to 6 (death).

*Cardiovascular risk factors* The MI controls answered questions about the cardiovascular risk factors hypertension (treatment yes/no) and diabetes (prevalence yes/no) at the time of examination. This information had already been collected from the OHCA survivors at the six-month TTM2-trial follow-up meeting.

### Statistical analyses

Descriptive statistics for continuous data are presented as means and standard deviations or medians and quartiles 1–3, depending on the distribution of the data. Binary and categorical data are presented as count and percentages. To explore the profile of OHCA survivors with major impairment *z* ≤ − 2 in at least one of six neuropsychological composite scores, these OHCA survivors were compared to the remaining OHCA survivors with Wilcoxon Mann–Whitney *U* test or Fisher’s exact test in a sub-group analysis.

Effect sizes on the *z*-score based neuropsychological composite scores were calculated with Cohen’s *d*, and reported as follows: 0.2–0.49 = Small/slight; 0.5–0.79 = Moderate; > 0.8 = Strong [28]. Further between-group differences on the neuropsychological composite scores were investigated with linear regression. For each cognitive domain, analyses were performed in three steps and reported as the mean difference (equal to the slope β). As step 1, unadjusted regressions were performed. To examine our hypothesis according to the pre-specified analysis plan, the same analyses were repeated but adjusted for level of education (university education, yes/no) and sex (male/female) as step 2. Additional linear regression models were calculated as step 3 to adjust for factors that could be associated with cognitive impairment; level of education, sex, anxiety and depression (HADS-A and HADS-D), fatigue (the five MFI-20 dimensions), insomnia (MISS), hypertension, and diabetes.

Associations between anxiety, depression, fatigue, insomnia, hypertension, diabetes, functional outcome, and neuropsychological composite scores were calculated with Spearman’s rho. Associations were reported as follows: 0.1–0.29 = Small/slight; 0.3–0.49 = Moderate; > 0.5 = Large/high [28].

All tests were two-tailed, and results were considered statistically significant at *p* < 0.05. Analyses were performed with R 3.6.3 (R Foundation for Statistical Computing, Vienna, Austria) and SPSS Statistics 27 (IBM Corp, Armonk, NY, USA).

## Results

### Participants

**Fig. 1:**
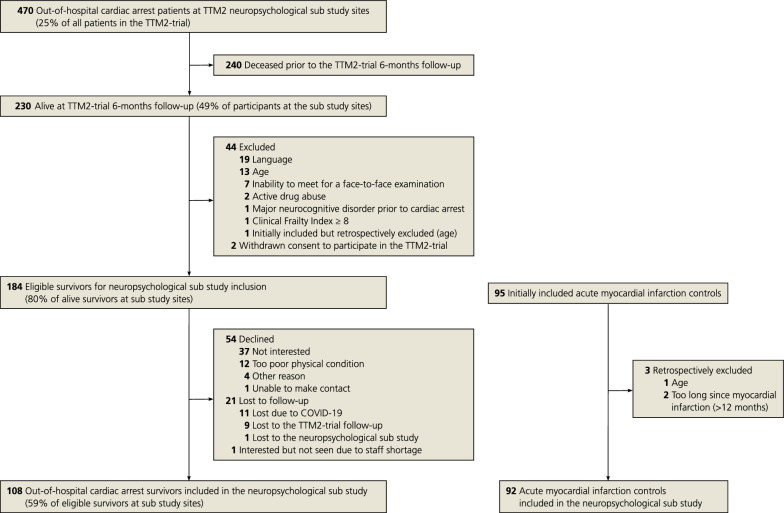
Flowchart of included out-of-hospital cardiac arrest survivors and acute myocardial infarction controls

A flow chart of the study inclusion can be found in Fig. 1. Of 184 eligible OHCA survivors, 108 were included in this study, together with 92 MI controls. The demographic and clinical characteristics of the included participants are reported in Table 1.

**Table 1:** Sociodemographic and medical background variables on all TTM2-survivors, included OHCA survivors, and included MI controls

	All TTM2 OHCA-survivors (*n* = 939)	Included OHCA survivors (*n* = 108)	Included MI controls (*n* = 92)
*Sociodemographic data*			
Age in years at time of examination, median (*Q*_1_–*Q*_3_)	60 (51–70)	62 (56–70)	65 (57–71)
Male, *n* (%)	787 (84)	95 (88)	82 (89)
*Highest attained level of education*			
No complete formal education, *n* (%)	32 (4)	2 (2)	0 (0)
Complete primary/lower secondary school, *n* (%)	250 (30)	33 (31)	16 (18)
Complete upper secondary school, *n* (%)	267 (33)	31 (29)	50 (54)
University-level education, with or without degree, *n* (%)	267 (33)	42 (38)	26 (28)
Pre-cardiac event workers,* n* (%)	438 (54)	64 (59)	57 (62)
Previous neurological disease, *n* (%)	60 (7)	8 (7)	4 (4)
*Cause of the initial cardiac event*			
STEMI, *n* (%)	388 (41)	43 (40)	54 (68)
Non-STEMI, *n* (%)	124 (13)	19 (17)	25 (32)
Arrhythmia due to cardiomyopathy or due to primary heart rhythm, *n* (%)	209 (22)	20 (18)	0 (0)
Other cardiac causes, *n* (%)	163 (18)	21 (20)	0 (0)
Other medical causes, *n* (%)	55 (6)	5 (5)	0 (0)
*Prehospital variables*			
Bystander-performed cardiopulmonary resuscitation, *n* (%)	796 (85)	94 (87)	n/a
First monitored rhythm shockable, *n* (%)	834 (89)	96 (89)	n/a
Time in minutes from arrest to sustained ROSC, median (*Q*_1_–*Q*_3_)	20 (14–30)	21 (14–30)	n/a
*In-hospital and rehabilitation data*			
Days at hospital, median (*Q*_1_–*Q*_3_)	15 (10–25)	15 (12–26)	5 (4–6)
Participation in rehabilitation interventions after cardiac event			
Cardiac rehabilitation, *n* (%)	232 (28)	24 (22)	58 (63)
Neurorehabilitation, *n* (%)	137 (16)	20 (19)	0 (0)
Other, *n* (%)	209 (22)	46 (43)	4 (4)
*At time of examination*			
Days from cardiac event until examination, median (*Q*_1_–*Q*_3_)	n/a	233 (213–287)	279 (241–323)
Living at home,* n* (%)	787 (96)	105 (97)	92 (100)
Hypertension, *n* (%)	584 (74)	69 (64)	71 (77)
Diabetes, *n* (%)	108 (13)	13 (12)	18 (20)
*Psychotropic drug use*			
Anxiolytics, *n* (%)	n/a	8 (7)	4 (4)
Antidepressants, *n* (%)	n/a	10 (9)	2 (2)
Sedatives/hypnotics, *n* (%)	n/a	14 (13)	3 (3)
Poor functional outcome (mRS 4–5),* n* (%)	56 (7)	4 (4)	n/a
Montreal Cognitive Assessment score < 26 indicating cognitive impairment,* n* (%)	309 (41)	37 (34)	n/a

Number of participants per site (OHCA/MI): Malmo, Sweden, *n* = 12/0; Lund, Sweden, *n* = 11/53; Helsingborg, Sweden, *n* = 11/0; Halmstad, Sweden, *n* = 5/0; Gothenburg, Sweden, *n* = 19/0; Aarhus, Denmark, *n* = 19/12; Basildon, the United Kingdom, *n* = 27/27; Cardiff, the United Kingdom, *n* = 4/0

TTM2, Targeted hypothermia versus targeted normothermia after out-of-hospital cardiac arrest trial; OHCA, out-of-hospital cardiac arrest; MI, myocardial infarction; *Q*_1_–*Q*_3_, quartile 1 to quartile 3; ROSC, return of spontaneous circulation; mRS, modified Rankin Scale

When comparing all 939 TTM2-survivors with the 108 included OHCA survivors, the median age was 60 vs 62, and 84% vs 88% were men. Of all TTM2-survivors, 33% had > 12 years of formal education as compared to 38% of included OHCA survivors. On the Montreal Cognitive Assessment (MoCA) that was used as a cognitive screening at six-months in the TTM2-trial, 41% of all TTM2-survivors and 34% of included OHCA survivors had scores < 26 indicating cognitive impairment [29]. Acute MI (STEMI and non-STEMI) was the cause of the initial cardiac arrest in 54% vs 57% of cases. For a detailed comparison, see Table 1.

### Descriptive outcome on the neuropsychological tests

**Fig. 2:**
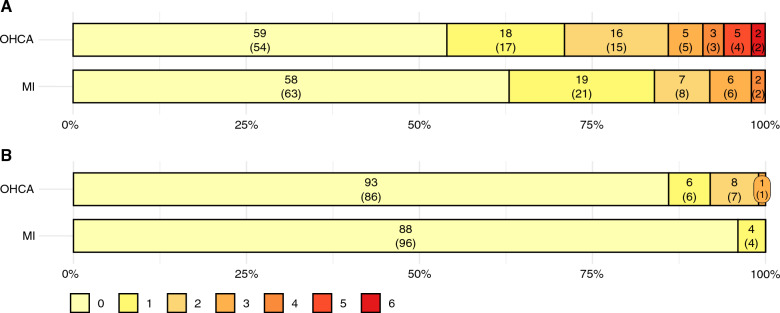
Number of cognitive domains (0–6) with cognitive impairment in of out-of-hospital cardiac arrest (OHCA) survivors and myocardial infarction (MI) controls, *n* (%). At least borderline–mild impairment (*z* ≤ -1) in A, major impairment (*z* ≤ -2) in B. Key in the bottom

Performance without cognitive impairment on all six composite scores occurred among 54% of OHCA survivors and 63% of MI controls (Fig. 2a). Scores indicating at least borderline–mild impairment were most common in one single composite score in both OHCA survivors (17%) and MI controls (21%). At least borderline–mild impairment on two or more of the composite scores occurred in 29% of OHCA survivors and 16% of MI controls. Major cognitive impairment in one or more composite scores was observed in 14% of OHCA survivors and 4% of MI controls, see Fig. 2b.

**Fig. 3:**
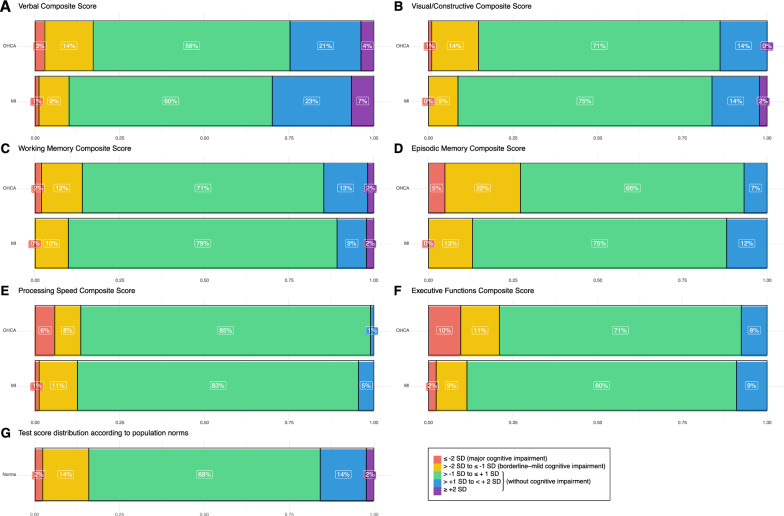
Result distribution on the neuropsychological composite scores (**a–f**) with of out-of-hospital cardiac arrest (OHCA) survivors and myocardial infarction (MI) controls, as well as assumed distribution in non-clinical groups according to population norms (**g**). Key with standard deviations (SD) on the lower right

The largest deficits, indicating at least borderline–mild impairment on the composite scores, were found in episodic memory (OHCA: 27%; MI: 13%) and executive functions (OHCA: 21%; MI: 11%), see Fig. 3a–f and Table 2. Major impairment was most frequently observed among OHCA survivors in executive functions (10%), processing speed (6%), and episodic memory (5%). Overall, MI controls performed similar to or better than the test score distribution according to population norms (Fig. 3g).

**Table 2:** Statistics on test performances in* z*-scores on the composite scores and associated neuropsychological tests

	*M* (*SD*)		Cohen’s *d*	Performance *z* ≤ − 1, *n* (%)		Performance *z* ≤ − 2, *n* (%)	
	OHCA	MI		OHCA	MI	OHCA	MI
**Verbal composite score**	**0.13 (1.09)**	**0.38 (1.02)**	− **0.24**	**18 (17)**	**9 (10)**	**3 (3)**	**1 (1)**
WAIS-IV Vocabulary	− 0.14 (1.02)	0.15 (0.91)	− 0.30	27 (26)	15 (16)	1 (1)	0 (0)
D-KEFS Verbal Fluency, letter fluency	0.14 (1.34)	0.23 (1.41)	− 0.07	26 (24)	21 (24)	8 (7)	7 (8)
D-KEFS Verbal Fluency, category fluency	0.40 (1.43)	0.76 (1.25)	− 0.27	23 (21)	8 (9)	5 (4)	2 (2)
**Visual/constructive composite score**	**0.01 (0.82)**	**0.22 (0.77)**	− **0.26**	**16 (15)**	**8 (9)**	**1 (1)**	**0 (0)**
WAIS-IV Block Design	0.14 (0.97)	0.24 (0.86)	− 0.11	17 (16)	10 (11)	1 (1)	1 (1)
WAIS-IV Matrix Reasoning	− 0.12 (0.95)	0.21 (0.93)	− 0.35	24 (22)	12 (13)	3 (3)	3 (3)
**Working memory composite score**	**0.00 (0.90)**	**0.06 (0.73)**	− **0.07**	**15 (14)**	**9 (10)**	**2 (2)**	**0 (0)**
WAIS-IV Digit Span	− 0.11 (0.92)	− 0.12 (0.81)	0.01	19 (18)	17 (19)	1 (1)	0 (0)
WMS-III Spatial Span	0.11 (1.18)	0.24 (0.99)	− 0.12	28 (26)	18 (20)	5 (5)	1 (1)
**Episodic memory composite score**	− **0.39 (0.95)**	− **0.07 (0.82)**	− **0.36**	**28 (27)**	**12 (13)**	**5 (5)**	**0 (0)**
RAVLT total recall	− 0.39 (1.31)	− 0.07 (1.14)	− 0.26	36 (34)	19 (21)	13 (12)	3 (3)
RAVLT delayed recall	− 0.34 (1.18)	− 0.08 (1.04)	− 0.24	30 (29)	16 (17)	10 (10)	2 (2)
WMS-III Logical Memory I	− 0.32 (1.13)	− 0.10 (1.03)	− 0.20	34 (32)	23 (25)	13 (12)	4 (4)
WMS-III Logical Memory II	0.01 (1.02)	0.24 (1.01)	− 0.23	22 (21)	15 (16)	5 (5)	2 (2)
BVMT-R total recall	− 0.92 (1.37)	− 0.46 (1.07)	− 0.37	53 (49)	32 (34)	21 (19)	6 (6)
BVMT-R delayed recall	− 0.51 (1.40)	0.02 (1.17)	− 0.41	40 (38)	20 (22)	14 (13)	5 (6)
**Processing speed composite score**	− **0.22 (1.09)**	− **0.10 (0.76)**	− **0.13**	**14 (14)**	**11 (12)**	**6 (6)**	**1 (1)**
TMT A	− 0.07 (1.88)	0.13 (1.03)	− 0.13	20 (18)	8 (9)	6 (5)	3 (3)
D-KEFS Color Word Interference Test, color naming	− 0.35 (1.01)	− 0.23 (0.94)	− 0.13	30 (28)	22 (25)	9 (8)	4 (5)
D-KEFS Color Word Interference Test, word reading	− 0.25 (0.92)	− 0.23 (0.84)	− 0.02	24 (23)	24 (27)	6 (6)	5 (6)
**Executive functions composite score**	− **0.30 (1.67)**	− **0.02 (0.86)**	− **0.21**	**22 (21)**	**10 (11)**	**10 (10)**	**2 (2)**
TMT B	− 1.03 (3.83)	− 0.39 (1.74)	− 0.22	35 (32)	25 (28)	23 (21)	10 (11)
D-KEFS Color Word Interference Test, inhibition	− 0.11 (1.11)	− 0.06 (1.20)	− 0.04	21 (19)	19 (21)	11 (10)	10 (11)
D-KEFS Color Word Interference Test, inhibition total errors	0.21 (0.95)	0.39 (0.67)	− 0.22	10 (10)	3 (3)	6 (6)	1 (1)

Bold text represent composite scores; regular text represent neuropsychological tests used for calculations of the associated composite scores.

Negative *z*-scores reflect worse scores than the population mean. *Z*-scores (normative *M* = 0, *SD* = 1) are adjusted for age (as well as for education on the TMT)

Negative values on Cohen’s *d* represent worse effects for the OHCA cohort than the MI cohort

On the BVMT-R Total recall and Delayed recall, standardized scores with the lowest value in the manual, corresponding to *z* < − 3, have been transformed to *z* − 4 to enable standardized analyses

Missing data were few (≤ 4) on all *z*-scores

OHCA, out-of-hospital cardiac arrest; MI, myocardial infarction; WAIS-IV, Wechsler Adult Intelligence Scale—Fourth Edition; D-KEFS, Delis-Kaplan Executive Function System; WMS-III, Wechsler Memory Scale—Third Edition; RAVLT, Rey Auditory Verbal Learning Test; BVMT-R, Brief Visuospatial Memory Test-Revised; TMT, Trail Making Test

On the individual neuropsychological tests used for composite score calculation, the greatest impairment amongst OHCA survivors was found on the episodic memory tests BVMT-R (total recall *z* ≤ − 1: 47%) and RAVLT (total recall *z* ≤ − 1: 34%), and the executive test TMT B (*z* ≤ − 1: 32%). See Table 2, and Additional file 1: Fig. S1 and Table S1.

In an exploratory subgroup analysis, OHCA survivors with major cognitive impairment in at least one of six composite scores (*n* = 15, 14%) had higher rates of diabetes (*p* = 0.03) and lower rates of bystander-performed cardiopulmonary resuscitation (*p* = 0.02) compared to the remaining included 93 OHCA survivors. OHCA survivors with major cognitive impairment had a longer intensive care unit (*p* = 0.001) and hospital (*p* < 0.001) stay. At the time of examination, they reported more symptoms of depression (*p* = 0.01) and fatigue (physical: *p* = 0.02; mental: *p* = 0.001; reduced activity: *p* < 0.001; reduced motivation: *p* = 0.002), and more frequently used psychotropic drugs (anxiolytics: *p* = 0.01; antidepressants: *p* = 0.004; sedatives/hypnotics: *p* = 0.02). Other sociodemographic, comorbidity, and prehospital variables were non-significant (Additional file 1: Table S2).

### Comparative analyses of the neuropsychological tests in OHCA survivors and MI controls

The unadjusted regressions showed that OHCA survivors performed significantly worse than MI controls in the cognitive domain of episodic memory (step 1; Table 3). Here, between-group differences were non-significant in the other five cognitive domains. Effect sizes ranged from negligible (processing speed, working memory) to slight (episodic memory, visual/constructive, verbal, executive functions) (Table 2).

**Table 3:** Analyses of *z*-score based regression models for cognitive domain differences between OHCA survivors and MI controls

	Step 1: unadjusted models		Step 2: models adjusted for level of education and sex		Step 3: models adjusted for level of education, sex, symptoms of anxiety, depression, fatigue, insomnia, diabetes, and hypertension	
	Mean difference (95% CI)	*p*	Mean difference (95% CI)	*p*	Mean difference (95% CI)	*p*
Verbal composite score	− 0.25 (− 0.54, 0.05)	0.10	− 0.34 (− 0.62, − 0.07)	0.02*	− 0.39 (− 0.68, − 0.10)	0.009**
Visual/constructive composite score	− 0.21 (− 0.44, 0.01)	0.06	− 0.26 (− 0.47, − 0.04)	0.02*	− 0.38 (− 0.61, − 0.16)	0.001**
Working memory composite score	− 0.06 (− 0.29, 0.17)	0.59	− 0.11 (− 0.33, 0.12)	0.36	− 0.11 (− 0.35, 0.14)	0.38
Episodic memory composite score	− 0.31 (− 0.57, − 0.06)	0.02*	− 0.37 (− 0.61, − 0.12)	0.004**	− 0.42 (− 0.67, − 0.17)	0.001**
Processing speed composite score	− 0.12 (− 0.39, 0.15)	0.37	− 0.14 (− 0.41, 0.13)	0.31	− 0.27 (− 0.55, 0.00)	0.05
Executive functions composite score	− 0.28 (− 0.67, 0.11)	0.15	− 0.31 (− 0.70, 0.08)	0.12	− 0.44 (− 0.82, − 0.06)	0.02*

Negative mean differences represent worse results on the composite scores for OHCA survivors than MI controls. Number of participants per analysis (step 1/step 2/step 3): Verbal composite score, *n* = 195/195/185; Visual/constructive composite score, *n* = 200/200/190; Working memory composite score, *n* = 200/200/190; Episodic memory composite score, *n* = 196/196/187; Processing speed composite score, *n* = 194/194/185; Executive functions composite score, *n* = 193/193/184

OHCA, out-of-hospital cardiac arrest; MI, myocardial infarction; CI, Confidence intervals

*Statistical significance *p* < 0.05

**Statistical significance *p* < 0.01

In the main, pre-specified regressions adjusted for level of education and sex, OHCA survivors performed worse than MI controls in three of six cognitive domains: episodic memory, verbal, and visual/constructive functions (step 2; Table 3).

In the regressions adjusted for level of education, sex, anxiety, depression, fatigue, insomnia, hypertension, and diabetes, OHCA survivors performed worse than MI controls in 4 of 6 cognitive domains, now including executive functions as well (step 3; Table 3).

### Descriptive outcome on the questionnaires

Levels of self-reported symptoms of anxiety, depression, fatigue, and insomnia are presented in Table 4. The proportion of results indicating possible anxiety (OHCA = 21%, MI = 26%) and depression (OHCA = 15%, MI = 17%) were similar between groups.

**Table 4:** Results on self-reported symptom questionnaires

	OHCA (*n* = 108)		MI (*n* = 92)	
	Median (*Q*_1_–*Q*_3_)	Possible clinical conditions, *n* (%)	Median (*Q*_1_–*Q*_3_)	Possible clinical conditions, *n* (%)
HADS anxiety subscale (*Min–Max* = 0–21)	3 (1–6)	16 (15)	4 (2–7)	16 (18)
HADS depression subscale (*Min–Max* = 0–21)	2 (1–4)	13 (12)	2 (1–6)	9 (10)
MFI-20 general fatigue subscale (*Min–Max* = 4–20)	10 (8–13)	n/a	11 (8–15)	n/a
MFI-20 physical fatigue subscale (*Min–Max* = 4–20)	11 (7–14)	n/a	10 (8–14)	n/a
MFI-20 Reduced Activity Subscale (*Min–Max* = 4–20)	10 (6–13)	n/a	9 (7–13)	n/a
MFI-20 reduced motivation subscale (*Min–Max* = 4–20)	8 (6–11)	n/a	9 (6–12)	n/a
MFI-20 mental fatigue subscale (*Min–Max* = 4–20)	8 (4–10)	n/a	8 (4–11)	n/a
MISS (*Min–Max* = 0–12)	3 (1–5)	24 (22)	4 (2–6)	28 (31)

Numeric low scores represent fewer symptoms on all questionnaires. Possible clinical conditions are defined as ≥ 8 on the HADS subscales and ≥ 6 on the MISS. Number of completed questionnaires (OHCA/MI): HADS, *n* = 107/91; MFI, *n* = 105/90; MISS, *n* = 107/91

OHCA, out-of-hospital cardiac arrest; MI, myocardial infarction; *Q*_1_–*Q*_3_, quartile 1 to quartile 3; HADS, Hospital Anxiety and Depression Scale; MFI-20, Multidimensional Fatigue Inventory; MISS, Minimal Insomnia Symptom Scale

### Associations between neuropsychological tests, questionnaires, cardiovascular risk factors, and functional outcome in OHCA survivors

*Emotional problems* Anxiety symptoms were slightly associated with worse executive functions (*r*_*s*_ = − 0.21, *p* = 0.01). Depressive symptoms were moderately associated with worse executive functions (*r*_*s*_ = − 0.37, *p* < 0.001) and slightly associated with worse processing speed (*r*_*s*_ = − 0.27, *p* = 0.01).

*Fatigue* General fatigue was slightly associated with worse executive functions (*r*_*s*_ = − 0.24, *p* = 0.01). Mental fatigue was slightly associated with worse episodic memory (*r*_*s*_ = − 0.21, *p* = 0.03) and executive functions (*r*_*s*_ = − 0.25, *p* = 0.01). Physical fatigue was slightly associated with worse processing speed (*r*_*s*_ = − 0.26, *p* = 0.01) and executive functions (*r*_*s*_ = − 0.24, *p* = 0.01).

*Insomnia* Insomnia symptoms were not significantly associated with neuropsychological test performance.

*Cardiovascular risk factors* Diabetes was slightly associated with worse working memory (*r*_*s*_ = − 0.18, *p* = 0.03), visual/constructive (*r*_*s*_ = − 0.23, *p* = 0.01) and executive functions (*r*_*s*_ = − 0.22, *p* = 0.02), while hypertension was not significantly associated with neuropsychological test performance. See Additional file 1: Table S3.

*Functional outcome* The mRS was moderately associated with verbal functions (*r*_*s*_ = − 0.35, *p* < 0.001), and slightly associated with episodic memory (*r*_*s*_ = − 0.29, *p* = 0.01), visual/constructive and executive functions (*r*_*s*_ = − 0.23, *p* = 0.02) (Additional file 1: Table S4).

## Discussion

In this study, we describe detailed information on neuropsychological outcome following adult OHCA in the late recovery phase at approximately 7 months post-arrest, and in relation to MI controls. In addition, we explore the relationship between cognition and anxiety, depression, fatigue, insomnia, hypertension, and diabetes. Our hypothesis, that OHCA survivors would perform significantly worse on the neuropsychological tests compared to a matched control group with acute MI, was upheld for the verbal, visual/constructive, and episodic memory domains when adjusting for educational attainment and sex.

Although exceptions with lower impairment rates exist [30], most previous research reports that about half of OHCA survivors have long-term cognitive impairment [4, 5]. In our data, this number was less in the late recovery phase with 29% having at least borderline–mild impairment in two or more neuropsychological composite scores. Since 14% had major impairment in at least one composite score, cognitive impairment in the current study was in general, mild. Major cognitive impairment was most frequent in the cognitive domains of episodic memory, processing speed, and executive functions. This finding is consistent with the literature [5].

In the subgroup analysis, a prolonged length of hospital stay and diabetes were more common among OHCA survivors with major cognitive impairment than remaining OHCA survivors. Some OHCA survivors may exhibit major cognitive impairment related to the hypoxic-ischemic brain injury caused by the OHCA, in addition to underlying cardiovascular burden. This study cannot discriminate between pre-arrest cognitive function in diabetes and possible exacerbation in case of OHCA, so additional research is required to investigate these findings. Furthermore, psychotropic drug use, which was more frequent in this subgroup analysis, as well as risk factors associated with post-intensive care syndrome could also have an impact on cognition following OHCA [31, 32]. We did not analyze the data based on temperature allocation in the current study, as pre-specified [14]. This approach is supported by the fact that there were no significant differences in hypothermia and normothermia for mortality and neurocognitive outcome in the main TTM2-trial, which included a larger number and proportion of eligible participants [13, 33].

When comparing groups, worse episodic memory performance among OHCA survivors was evident in all analyses and worse than population norms, suggesting clinical relevance. This was also the most manifest difference between the OHCA and MI cohorts on effect size measures. The smaller group of OHCA survivors with major cognitive impairment might be driving the group-effect. Although just a slight effect when comparing the cohorts according to benchmarks [28], it could be considered large for those affected by impairment. A larger number of OHCA survivors had cognitive impairment compared to normative data on processing speed and executive measures, but this was surprisingly not reflected as significant between-group differences in the pre-specified adjusted regression models. When adjusting for associated factors (step 3), OHCA survivors performed worse than MI controls on the executive functions composite score. In contrast, the worse verbal and visual/constructive performance among OHCA survivors may not be of particular clinical significance since, overall, both groups performed similarly to population norms in these domains.

A recent study comparing cognitive outcome following OHCA and MI reported approximately six times higher rates of cognitive impairment after OHCA than MI [34], a more distinct between-group difference than in our study. This prior study was performed close to hospital discharge and hence closer to the cardiac event than the current study. Indeed, a prevalence of cognitive impairment up to 80–100% has been reported in the early stages of recovery after OHCA [35, 36]. Like other forms of acquired brain injury, the greatest cognitive improvement occurs during the first three months post-arrest [37]. It is probable that many OHCA survivors have an early cognitive impairment but that several survivors have improved in the late recovery phase. A previous study from our group conducted at approximately six months post-OHCA found deficits in memory and executive functions that were similar between OHCA survivors and MI controls, but processing speed was worse among OHCA survivors [10]. This differs from the worse memory scores among OHCA survivors, compared to MI controls, in the present study. The difference might be an effect of the improved general sensitivity of the tests in this study.

We also aimed to investigate the relationship between post-arrest cognitive functioning and associated factors. The proportion of results representing possible anxiety (15%) and depression (12%) among our OHCA survivors were somewhat lower than the pooled six-months prevalence in a recent meta-analysis (34% and 17%, respectively), but still higher than the estimated prevalence in the general population [11]. Our finding that cognitive impairment was associated with symptoms of anxiety, depression, and fatigue following OHCA is in line with previous research [8, 38]. Worse executive functions were particularly related to emotional problems. The relationship between emotional problems and the neuropsychological test profile is in accordance with what has been observed in mood disorders without OHCA [39, 40]. Furthermore, mental fatigue was associated with deficits in episodic memory and executive functions. This is in agreement with some earlier acquired brain injury studies [41, 42]. Unlike hypertension, diabetes was significantly related to cognitive performance, which is consistent with a growing body of evidence indicating that diabetes impairs cognition over and above the burden of cerebrovascular pathology [43].

The interplay between cognition and the associated factors is complex. Mood disorders may partly predispose individuals to an OHCA by contributing to risk factors such as cardiovascular disease and diabetes [44], which in turn are associated with a higher probability of cognitive decline [45, 46]. Following OHCA, cognitive impairment is associated with depressive symptoms [8]. Meanwhile, post-arrest patient-reported cognitive complaints may represent emotional problems rather than cognitive decline [47]. Sleep disturbances might be symptoms of depression, and both could lead to sub-optimal cognitive performance [48], so awareness of all these interactions is essential to provide appropriate treatment.

The concise, clinician-reported mRS is currently recommended to assess functional outcome after OHCA in clinical trials [27, 49]. Earlier studies have reported correlations between clinician-reported outcome and performance-based cognitive tests such as neuropsychological tests [47, 50]. The measures are not directly comparable [49, 51], as again reflected by our results. Correspondingly, neuropsychological tests are sensitive for detecting clinical signs of hypoxic-ischemic brain injury that global outcome measures such as the mRS may overlook.

We used composite scores to form the cognitive domains, a common practice in earlier neuropsychological studies on OHCA survivors [52–54]. Composite scores reduce the number of variables but also decrease granularity, as the individual neuropsychological tests measure different sub-components of the same overall cognitive domain. For instance, even though we used composite classifications and tests previously administered to this population [53, 55–57], 21% of OHCA survivors performed to a level consistent with major cognitive impairment on the TMT B while only 10% obtained these scores on the D-KEFS CWIT 3. Cognitive flexibility (TMT B) may be more impaired than inhibition (CWIT 3) among OHCA survivors. Since TMT B and CWIT 3 both form the executive functions composite score, it is possible that the inclusion of the CWIT in the test battery has obscured OHCA-related executive impairment in our study.

As for clinical importance, the detailed data from this study may be used to guide measurement selection in clinical practice and research, considering the growing need for standardization in measures of neurocognitive function following OHCA [5, 58]. For example, the BVMT-R, RAVLT, and TMT were sensitive tests and could be candidate measures for a future neuropsychological test battery post-arrest. Moreover, our results highlight that cognitive, emotional, and fatigue screenings are all vital to identify impairment and distress in routine post-arrest follow-up, in line with current European guidelines [59]. Individuals with indicated impairment might benefit from a comprehensive evaluation with more sensitive measures. If necessary, treatment of emotional problems and fatigue management should be considered [60, 61], or patient-centered cognitive rehabilitation in memory, executive functioning, and attention/processing speed. These cognitive domains were the most affected in our study and seem to be predominantly impaired in the early stages of post-arrest recovery as well [37, 62]. Future work may additionally address the interplay between cognitive outcome, emotional problems, fatigue, cardiovascular risk factors, neuroimaging, and overall functional outcome in everyday life. Cognitive change over time should also be further investigated.

As for strengths of the study, we selected neuropsychological tests with high sensitivity when administered to OHCA survivors in previous research, added them to form an extensive test battery, and collected information on emotional problems, fatigue, insomnia, and cardiovascular risk factors. This generated a detailed outline of the post-OHCA neuropsychological profile, compared to the much used but less sensitive screening measures. A relatively large number of OHCA survivors and a matched control group were included, enabling comparisons with the general population through population norms and a group with similar cardiovascular risk factors.

There were important limitations to this study. Since only arrests of presumed cardiac or unknown causes were included in the TTM2-trial, our participants are not representative of all OHCA survivors. This could hamper study generalizability. As a sub-study of a large trial, we were able to compare our included OHCA survivors with all TTM2-survivors, increasing the internal validity of our study. Fewer included survivors had an indicated cognitive impairment on the MoCA than all TTM2-survivors, suggesting that OHCA survivors with cognitive impairment may be underrepresented in this study. Moreover, 57% of the OHCA survivors had acute MI as cause of arrest, while the MI controls had all suffered acute MI. We did also not accomplish a perfect 1:1 OHCA–MI ratio since the COVID-19 pandemic halted clinical research at all study sites at some time-points, and the matching of MI controls was based by country and not by site due to pragmatic reasons. However, the OHCA and MI cohorts were relatively equivalent in size and demographic variables. In addition, there are different standards of life support interventions depending on country of OHCA, and our results may only be translated to countries with similar intervention traditions as the three countries in our study. Another limitation is not having information on premorbid cognitive status. It is particularly difficult to compare our binary classifications of cognitive impairment with population norms since we do not have population-based information on between-test correlations when involving multiple cognitive domains; some results need to be interpreted with caution due to multiple comparisons. Whilst many participants performed without impairment, we cannot exclude that some impaired scores represent low premorbid capacities. We however used level of education as a proxy for premorbid cognitive functioning in the regression analyses, as educational duration is positively correlated with premorbid intellectual capacity [63]. Most of the individual neuropsychological scores, and all composite scores since they lack prior validation, do not have established anchor-based values for minimally important differences. Relatedly, the criterion for cognitive impairment varies between studies [5, 64]. To mitigate this, we have described cognitive impairment according to established classifications [23], and reported raw scores to facilitate comparability between studies.

## Conclusions

Cognitive impairment assessed by neuropsychological tests was generally mild among OHCA survivors in our study population, but could in some cases be severe and extend over multiple cognitive domains. Our hypothesis that OHCA survivors would perform worse than MI controls was confirmed. Diabetes and symptoms of anxiety, depression, and fatigue were associated with worse cognitive performance among the OHCA survivors. The most sensitive tests used in this detailed examination could guide future assessments in both clinical practice and research settings.

## Supplementary Information

**Additional file 1. Figure S1**. Result distribution on the neuropsychological tests. **Table S1**. Raw and standardized scores on the neuropsychological measures used for cognitive domain calculation. **Table S2**. Exploratory analyses on demographic and medical background variables for survivors with major cognitive impairment and survivors without major cognitive impairment. **Table S3**. Correlation matrix on neuropsychological composite scores and emotional problems, fatigue, and cardiovascular risk factors. **Table S4**. Spearman associations for neuropsychological composite scores and modified Rankin Scale scores.

## Data Availability

The data that will support the findings of this study are available from the TTM2-trial steering group, but restrictions apply to the availability of these data, and so are not publicly available. Data are however available from the authors upon reasonable request and with permission of the TTM2-trial steering group.

## Abbreviations

BVMT-R: Brief Visuospatial Memory Test-Revised
CWIT: Color-Word Interference Test
D-KEFS: Delis-Kaplan Executive Function System
HADS: Hospital Anxiety and Depression Scale
MD: Mean difference
MI: Myocardial infarction
MFI-20: Multidimensional Fatigue Inventory
MISS: Minimal Insomnia Symptom Scale
MoCA: Montreal Cognitive Assessment
mRS: Modified Rankin Scale
OHCA: Out-of-hospital cardiac arrest
RAVLT: Rey Auditory Verbal Learning Test
STEMI: ST-elevation myocardial infarction
TMT: Trail Making Test
TTM2: Targeted hypothermia versus targeted normothermia after out-of-hospital cardiac arrest trial
WAIS-IV: Wechsler Adult Intelligence Scale—Fourth Edition
WMS-III: Wechsler Memory Scale—Third Edition
