# Correction: Neuropsychological outcome after cardiac arrest: results from a sub-study of the targeted hypothermia versus targeted normothermia after out-of-hospital cardiac arrest (TTM2) trial

**DOI:** 10.1186/s13054-026-06188-2

**Published:** 2026-07-29

**Authors:** Erik Blennow Nordström, Susanna Vestberg, Lars Evald, Marco Mion, Magnus Segerström, Susann Ullén, John Bro‑Jeppesen, Hans Friberg, Katarina Heimburg, Anders M. Grejs, Thomas R. Keeble, Hans Kirkegaard, Hanna Ljung, Sofia Rose, Matthew P. Wise, Christian Rylander, Johan Unden, Niklas Nielsen, Tobias Cronberg, Gisela Lilja

**Affiliations:** 1 Neurology, Department of Clinical Sciences Lund, Skane University Hospital, Lund University, Lund, Sweden; 2 Department of Psychology, Lund University, Lund, Sweden; 3 Hammel Neurorehabilitation Centre and University Research Clinic, Hammel, Denmark; 4 Essex Cardiothoracic Centre, Mid and South Essex NHS Foundation Trust, Basildon, UK; 5 Medical Technology Research Centre, Anglia Ruskin School of Medicine, Chelmsford, UK; 6 Department of Neurology, Department of Cardiology, Sahlgrenska University Hospital, Gothenburg, Sweden; 7 Clinical Studies Sweden – Forum South, Skane University Hospital, Lund, Sweden; 8 Department of Cardiology, Aarhus University Hospital, Aarhus, Denmark; 9 Intensive and Perioperative Care, Department of Clinical Sciences Lund, Skane University Hospital, Lund University, Malmo, Sweden; 10 Department of Intensive Care Medicine, Department of Clinical Medicine, Aarhus University Hospital and Aarhus University, Aarhus, Denmark; 11 Research Centre for Emergency Medicine, Emergency Department and Department of Clinical Medicine, Aarhus University Hospital and Aarhus University, Aarhus, Denmark; 12 Clinical Psychology, Cardiff and Vale University Health Board, NHS Wales, Cardiff, UK; 13 Adult Critical Care, University Hospital of Wales, Cardiff, UK; 14 Anaesthesiology and Intensive Care Medicine, Department of Surgical Sciences, Uppsala University, Uppsala, Sweden; 15 Operation and Intensive Care, Hallands Hospital Halmstad, Halmstad, Sweden; 16 Anesthesiology and Intensive Care, Department of Clinical Sciences Lund, Helsingborg Hospital, Lund University, Lund, Sweden

**Correction: Critical Care (2023) 27:328**

10.1186/s13054-023-04617-0

Following publication of the original article [1], the authors identified errors in Tables 2 and 4, certain parts referring to Tables 2 and 4 in the Discussion section and in the Supplementary Fig. S1 in the Additional file 1. Both the incorrect and correct information is given hereafter. This does not affect the primary outcomes of the article.

The incorrect Table 2:

**Table 2:** Statistics on test performances in* z*-scores on the composite scores and associated neuropsychological tests

	*M* (*SD*)		Cohen’s *d*	Performance *z* ≤ - 1, *n* (%)		Performance *z* ≤ - 2, *n* (%)	
	OHCA	MI		OHCA	MI	OHCA	MI
**Verbal composite score**	**0.13 (1.09)**	**0.38 (1.02)**	**–0.24**	**18 (17)**	**9 (10)**	**3 (3)**	**1 (1)**
WAIS-IV Vocabulary	–0.14 (1.02)	0.15 (0.91)	–0.30	17 (16)	4 (4)	0 (0)	0 (0)
D-KEFS Verbal Fluency, letter fluency	0.14 (1.34)	0.23 (1.41)	–0.07	20 (19)	16 (17)	4 (4)	4 (4)
D-KEFS Verbal Fluency, category fluency	0.40 (1.43)	0.76 (1.25)	–0.27	14 (13)	6 (7)	3 (3)	1 (1)
**Visual/constructive composite score**	**0.01 (0.82)**	**0.22 (0.77)**	**–0.26**	**16 (15)**	**8 (9)**	**1 (1)**	**0 (0)**
WAIS-IV Block Design	0.14 (0.97)	0.24 (0.86)	–0.11	11 (10)	3 (3)	1 (1)	1 (1)
WAIS-IV Matrix Reasoning	–0.12 (0.95)	0.21 (0.93)	–0.35	13 (12)	7 (8)	1 (1)	0 (0)
**Working memory composite score**	**0.00 (0.90)**	**0.06 (0.73)**	**–0.07**	**15 (14)**	**9 (10)**	**2 (2)**	**0 (0)**
WAIS-IV Digit Span	–0.11 (0.92)	–0.12 (0.81)	0.01	12 (11)	8 (9)	0 (0)	0 (0)
WMS-III Spatial Span	0.11 (1.18)	0.24 (0.99)	–0.12	11 (10)	7 (8)	3 (3)	0 (0)
**Episodic memory composite score**	**–0.39 (0.95)**	**–0.07 (0.82)**	**–0.36**	**28 (27)**	**12 (13)**	**5 (5)**	**0 (0)**
RAVLT total recall	–0.39 (1.31)	–0.07 (1.14)	–0.26	36 (34)	19 (21)	13 (12)	3 (3)
RAVLT delayed recall	–0.34 (1.18)	–0.08 (1.04)	–0.24	30 (29)	16 (17)	10 (10)	2 (2)
WMS-III Logical Memory I	–0.32 (1.13)	–0.10 (1.03)	–0.20	26 (24)	15 (16)	7 (6)	4 (4)
WMS-III Logical Memory II	0.01 (1.02)	0.24 (1.01)	–0.23	15 (14)	10 (11)	4 (4)	0 (0)
BVMT-R total recall	–0.92 (1.37)	–0.46 (1.07)	–0.37	51 (47)	29 (32)	17 (16)	6 (7)
BVMT-R delayed recall	–0.51 (1.40)	0.02 (1.17)	–0.41	39 (37)	18 (19)	14 (13)	5 (5)
**Processing speed composite score**	**–0.22 (1.09)**	**–0.10 (0.76)**	**–0.13**	**14 (14)**	**11 (12)**	**6 (6)**	**1 (1)**
TMT A	–0.07 (1.88)	0.13 (1.03)	–0.13	20 (19)	8 (9)	6 (6)	3 (3)
D-KEFS Color Word Interference Test, color naming	–0.35 (1.01)	–0.23 (0.94)	–0.13	19 (18)	18 (20)	7 (7)	3 (3)
D-KEFS Color Word Interference Test, word reading	–0.25 (0.92)	–0.23 (0.84)	–0.02	18 (17)	9 (10)	5 (5)	2 (2)
**Executive functions composite score**	**–0.30 (1.67)**	**–0.02 (0.86)**	**–0.21**	**22 (21)**	**10 (11)**	**10 (10)**	**2 (2)**
TMT B	–1.03 (3.83)	–0.39 (1.74)	–0.22	34 (32)	25 (28)	23 (21)	10 (11)
D-KEFS Color Word Interference Test, inhibition	–0.11 (1.11)	–0.06 (1.20)	–0.04	17 (16)	17 (19)	10 (9)	6 (7)
D-KEFS Color Word Interference Test, inhibition total errors	0.21 (0.95)	0.39 (0.67)	–0.22	10 (10)	3 (3)	6 (6)	1 (1)

Bold text represent composite scores; regular text represent neuropsychological tests used for calculations of the associated composite scores. Negative *z*-scores reflect worse scores than the population mean. *Z*-scores (normative *M* = 0, *SD* = 1) are adjusted for age (as well as for education on the TMT). Negative values on Cohen’s *d* represent worse effects for the OHCA cohort than the MI cohort. On the BVMT-R Total recall and Delayed recall, standardized scores with the lowest value in the manual, corresponding to *z* < - 3, have been transformed to *z* - 4 to enable standardized analyses. Missing data were few (≤ 4) on all *z*-scores

OHCA, out-of-hospital cardiac arrest; MI, myocardial infarction; WAIS-IV, Wechsler Adult Intelligence Scale—Fourth Edition; D-KEFS, Delis-Kaplan Executive Function System; WMS-III, Wechsler Memory Scale—Third Edition; RAVLT, Rey Auditory Verbal Learning Test; BVMT-R, Brief Visuospatial Memory Test-Revised; TMT, Trail Making Test

The correct Table 2:

**Table 2:** Statistics on test performances in *z*-scores on the composite scores and associated neuropsychological tests

	M (SD)		Cohen’s d	Performance z ≤-1, *n* (%)		Performance z ≤-2, *n* (%)	
	OHCA	MI		OHCA	MI	OHCA	MI
**Verbal composite score**	**0.13 (1.09)**	**0.38 (1.02)**	**-0.24**	**18 (17)**	**9 (10)**	**3 (3)**	**1 (1)**
WAIS-IV vocabulary	-0.14 (1.02)	0.15 (0.91)	-0.30	27 (26)	15 (16)	1 (1)	0 (0)
D-KEFS verbal fluency, letter fluency	0.14 (1.34)	0.23 (1.41)	-0.07	26 (24)	21 (24)	8 (7)	7 (8)
D-KEFS verbal fluency, category fluency	0.40 (1.43)	0.76 (1.25)	-0.27	23 (21)	8 (9)	5 (4)	2 (2)
**Visual/constructive composite score**	**0.01 (0.82)**	**0.22 (0.77)**	**-0.26**	**16 (15)**	**8 (9)**	**1 (1)**	**0 (0)**
WAIS-IV block design	0.14 (0.97)	0.24 (0.86)	-0.11	17 (16)	10 (11)	1 (1)	1 (1)
WAIS-IV matrix reasoning	-0.12 (0.95)	0.21 (0.93)	-0.35	24 (22)	12 (13)	3 (3)	3 (3)
**Working memory composite score**	**0.00 (0.90)**	**0.06 (0.73)**	**-0.07**	**15 (14)**	**9 (10)**	**2 (2)**	**0 (0)**
WAIS-IV digit span	-0.11 (0.92)	-0.12 (0.81)	0.01	19 (18)	17 (19)	1 (1)	0 (0)
WMS-III spatial span	0.11 (1.18)	0.24 (0.99)	-0.12	28 (26)	18 (20)	5 (5)	1 (1)
**Episodic memory composite score**	**-0.39 (0.95)**	**-0.07 (0.82)**	**-0.36**	**28 (27)**	**12 (13)**	**5 (5)**	**0 (0)**
RAVLT total recall	-0.39 (1.31)	-0.07 (1.14)	-0.26	36 (34)	19 (21)	13 (12)	3 (3)
RAVLT delayed recall	-0.34 (1.18)	-0.08 (1.04)	-0.24	30 (29)	16 (17)	10 (10)	2 (2)
WMS-III logical memory I	-0.32 (1.13)	-0.10 (1.03)	-0.20	34 (32)	23 (25)	13 (12)	4 (4)
WMS-III logical memory II	0.01 (1.02)	0.24 (1.01)	-0.23	22 (21)	15 (16)	5 (5)	2 (2)
BVMT-R total recall	-0.92 (1.37)	-0.46 (1.07)	-0.37	53 (49)	32 (34)	21 (19)	6 (6)
BVMT-R delayed recall	-0.51 (1.40)	0.02 (1.17)	-0.41	40 (38)	20 (22)	14 (13)	5 (6)
**Processing speed composite score**	**-0.22 (1.09)**	**-0.10 (0.76)**	**-0.13**	**14 (14)**	**11 (12)**	**6 (6)**	**1 (1)**
TMT A	-0.07 (1.88)	0.13 (1.03)	-0.13	20 (18)	8 (9)	6 (5)	3 (3)
D-KEFS color word interference test, color naming	-0.35 (1.01)	-0.23 (0.94)	-0.13	30 (28)	22 (25)	9 (8)	4 (5)
D-KEFS color word interference test, word reading	-0.25 (0.92)	-0.23 (0.84)	-0.02	24 (23)	24 (27)	6 (6)	5 (6)
**Executive functions composite score**	**-0.30 (1.67)**	**-0.02 (0.86)**	**-0.21**	**22 (21)**	**10 (11)**	**10 (10)**	**2 (2)**
TMT B	-1.03 (3.83)	-0.39 (1.74)	-0.22	35 (32)	25 (28)	23 (21)	10 (11)
D-KEFS color word interference test, inhibition	-0.11 (1.11)	-0.06 (1.20)	-0.04	21 (19)	19 (21)	11 (10)	10 (11)
D-KEFS color word interference test, inhibition total errors	0.21 (0.95)	0.39 (0.67)	-0.22	10 (10)	3 (3)	6 (6)	1 (1)

OHCA, out-of-hospital cardiac arrest; MI, myocardial infarction; WAIS-IV, Wechsler Adult Intelligence Scale – Fourth Edition; D-KEFS, Delis-Kaplan Executive Function System; WMS-III, Wechsler Memory Scale – Third Edition; RAVLT, Rey Auditory Verbal Learning Test; BVMT-R, Brief Visuospatial Memory Test-Revised; TMT, Trail Making Test

Bold text represent composite scores; regular text represent neuropsychological tests used for calculations of the associated composite scores

Negative *z*-scores reflect worse scores than the population mean. *Z*-scores (normative *M* = 0, *SD* = 1) are adjusted for age (as well as for education on the TMT)

Negative values on Cohen’s *d* represent worse effects for the OHCA cohort than the MI cohort

On the BVMT-R Total recall and Delayed recall, standardized scores with the lowest value in the manual, corresponding to *z* <-3, have been transformed to *z* -4 to enable standardized analyses

Missing data were few (≤ 4) on all *z*-scores

The incorrect Table 4:

**Table 4:** Results on self-reported symptom questionnaires

	OHCA (*n* = 108)		MI (*n* = 92)	
	Median (*Q*_1_-*Q*_3_)	Possible clinical conditions, *n* (%)	Median (*Q*_1_-*Q*_3_)	Possible clinical conditions, *n* (%)
HADS anxiety subscale (*Min-Max* = 0-21)	3 (1-6)	22 (21)	4 (2-7)	24 (26)
HADS depression subscale (*Min-Max* = 0-21)	2 (1-4)	16 (15)	2 (1-6)	16 (17)
MFI-20 general fatigue subscale (*Min-Max* = 4-20)	10 (8-13)	n/a	11 (8-15)	n/a
MFI-20 physical fatigue subscale (*Min-Max* = 4-20)	11 (7-14)	n/a	10 (8-14)	n/a
MFI-20 Reduced Activity Subscale (*Min-Max* = 4-20)	10 (6-13)	n/a	9 (7-13)	n/a
MFI-20 reduced motivation subscale (*Min-Max* = 4-20)	8 (6-11)	n/a	9 (6-12)	n/a
MFI-20 mental fatigue subscale (*Min-Max* = 4-20)	8 (4-10)	n/a	8 (4-11)	n/a
MISS (*Min-Max* = 0-12)	3 (1-5)	24 (22)	4 (2-6)	28 (31)

Numeric low scores represent fewer symptoms on all questionnaires. Possible clinical conditions are defined as ≥ 8 on the HADS subscales and ≥ 6 on the MISS. Number of completed questionnaires (OHCA/MI): HADS, *n* = 107/91; MFI, *n* = 105/90; MISS, *n* = 107/91

OHCA, out-of-hospital cardiac arrest; MI, myocardial infarction; *Q*_1_-*Q*_3_, quartile 1 to quartile 3; HADS, Hospital Anxiety and Depression Scale; MFI-20, Multidimensional Fatigue Inventory; MISS, Minimal Insomnia Symptom Scale

The correct Table 4:

**Table 4:** Results on self-reported symptom questionnaires

	OHCA (*n* = 108)		MI (*n* = 92)	
	Median (Q_1_–Q_3_)	Possible clinical conditions, *n* (%)	Median (Q_1_–Q_3_)	Possible clinical conditions, *n* (%)
HADS anxiety subscale (*Min–Max* = 0–21)	3 (1–6)	16 (15)	4 (2–7)	16 (18)
HADS depression subscale (*Min–Max* = 0–21)	2 (1–4)	13 (12)	2 (1–6)	9 (10)
MFI-20 general fatigue subscale (*Min–Max* = 4–20)	10 (8–13)	n/a	11 (8–15)	n/a
MFI-20 physical fatigue subscale (*Min–Max* = 4–20)	11 (7–14)	n/a	10 (8–14)	n/a
MFI-20 reduced activity subscale (*Min–Max* = 4–20)	10 (6–13)	n/a	9 (7–13)	n/a
MFI-20 reduced motivation subscale (*Min–Max* = 4–20)	8 (6–11)	n/a	9 (6–12)	n/a
MFI-20 mental fatigue subscale (*Min–Max* = 4–20)	8 (4–10)	n/a	8 (4–11)	n/a
MISS (*Min–Max* = 0–12)	3 (1–5)	24 (22)	4 (2–6)	28 (31)

OHCA, out-of-hospital cardiac arrest; MI, myocardial infarction; *Q*_1_–*Q*_3_, quartile 1 to quartile 3; HADS, Hospital anxiety and depression scale; MFI-20, Multidimensional fatigue inventory; MISS, Minimal insomnia symptom scale

Numeric low scores represent fewer symptoms on all questionnaires

Possible clinical conditions are defined as ≥ 8 on the HADS subscales and ≥ 6 on the MISS

Number of completed questionnaires (OHCA/MI): HADS, *n =* 107/91; MFI, *n =* 105/90; MISS, *n =* 107/91

The sentence in the Discussion section originally reads:

The proportion of results representing possible anxiety (21%) and depression (15%) among our OHCA survivors were somewhat lower than the pooled six-months prevalence in a recent meta-analysis (34% and 17%, respectively), but still higher than the estimated prevalence in the general population [11].

The sentence in the Discussion section currently reads:

The proportion of results representing possible anxiety (15%) and depression (12%) among our OHCA survivors were somewhat lower than the pooled six-months prevalence in a recent meta-analysis (34% and 17%, respectively), but still higher than the estimated prevalence in the general population [11].

The sentence in the Discussion section originally reads:

For instance, even though we used composite classifications and tests previously administered to this population [53, 55–57], 21% of OHCA survivors performed to a level consistent with major cognitive impairment on the TMT B while only 9% obtained these scores on the D-KEFS CWIT 3.

The sentence in the Discussion section currently reads:

For instance, even though we used composite classifications and tests previously administered to this population [53, 55–57], 21% of OHCA survivors performed to a level consistent with major cognitive impairment on the TMT B while only 10% obtained these scores on the D-KEFS CWIT 3.

The correct Supplementary Fig. S1 in the Additional file 1 is included in this correction article.

Tables 2 and 4, certain parts referring to Tables 2 and 4 in the Discussion section and the Supplementary Fig. S1 in the Additional file 1 have been updated in this correction article and the original article [1] has been corrected.

## Supplementary Information

Below is the link to the electronic supplementary material.

Supplementary Material 1.
