# Supplementary material for: Neuropsychological outcome after cardiac arrest: results from a sub-study of the targeted hypothermia versus targeted normothermia after out-of-hospital cardiac arrest (TTM2) trial
Source: Crit Care. 2023 Aug 26;27:328. doi: 10.1186/s13054-023-04617-0 (PMC10463667; doi:10.1186/s13054-023-04617-0)
Supplement: Supplementary file 1 — Additional file 1. Figure S1. Result distribution on the neuropsychological tests. Table S1. Raw and standardized scores on the neuropsychological measures used for cognitive domain calculation. Table S2. Exploratory analyses on demographic and medical background variables for survivors with major cognitive impairment and survivors without major cognitive impairment. Table S3. Correlation matrix on neuropsychological composite scores and emotional problems, fatigue, and cardiovascular risk factors. Table S4. Spearman associations for neuropsychological composite scores and modified Rankin Scale scores. [file 13054_2023_4617_MOESM1_ESM.pdf]

Additional file 1

Additional figure and tables

Neuropsychological outcome after cardiac arrest: Results from a sub-study of the Targeted hypothermia versus targeted normothermia after out-of-hospital cardiac arrest (TTM2) trial

Table of contents

Supplementary Figure

Supplementary Figure 1. Result distribution on the neuropsychological tests. *Page 2*

Supplementary Tables

Supplementary Table 1. Raw and standardized scores on the neuropsychological measures used for cognitive domain calculation. *Page 3*

Supplementary Table 2. Exploratory analyses on demographic and medical background variables for survivors with major cognitive impairment and survivors without major cognitive impairment. *Page 7*

Supplementary Table 3. Correlation matrix on neuropsychological composite scores and emotional problems, fatigue, and cardiovascular risk factors. *Page 9*

Supplementary Table 4. Spearman associations for neuropsychological composite scores and modified Rankin Scale scores. *Page 11*

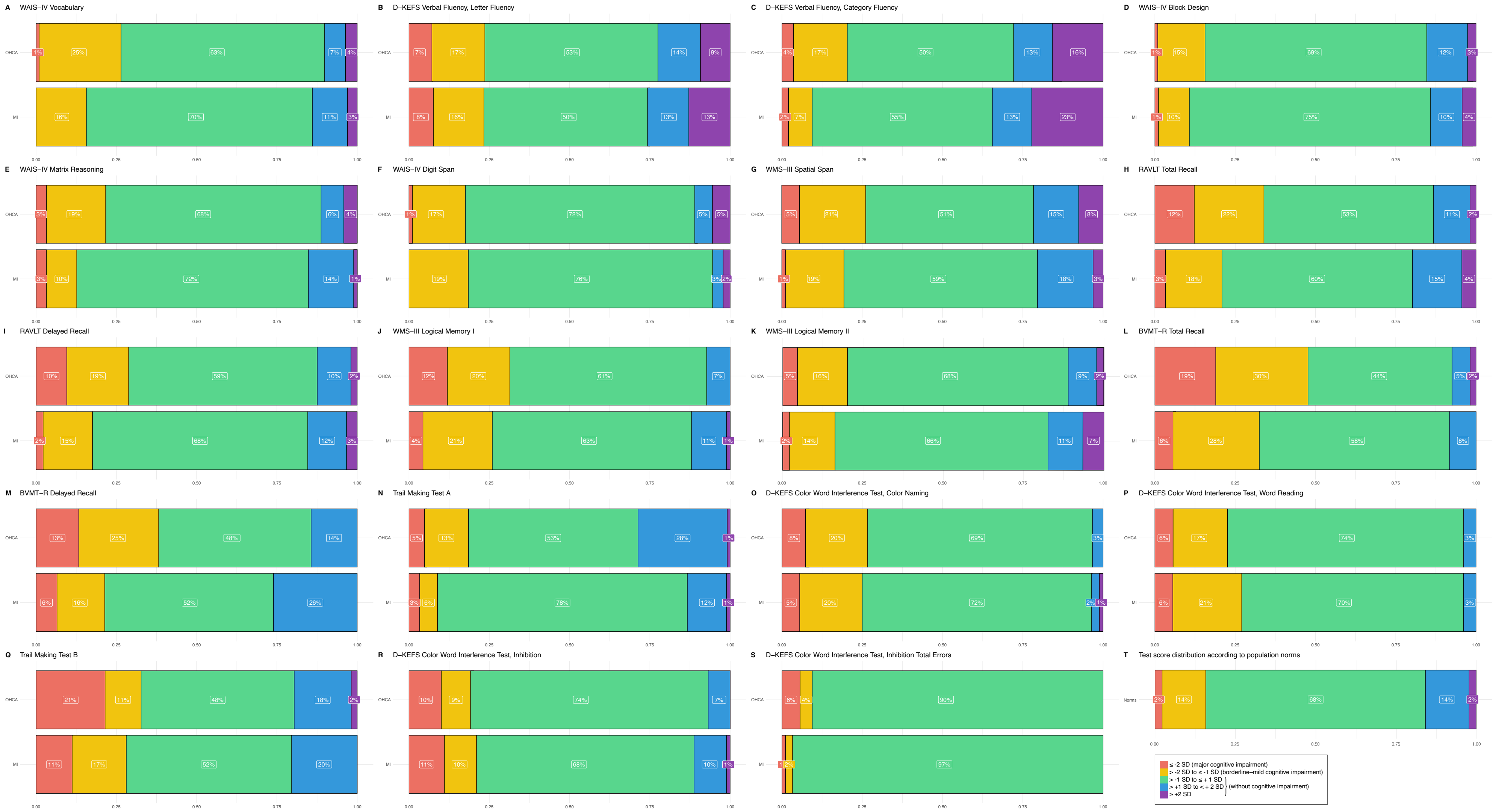

Supplementary Figure 1. **Result distribution on the neuropsychological tests (A–S) with of out-of-hospital cardiac arrest (OHCA) survivors and myocardial infarction (MI) controls, as well as assumed distribution in non-clinical groups according to population norms (T). Key with standard deviations (SD) on the lower right.**

Supplementary Table 1. **Raw and standardized scores on the neuropsychological measures used for cognitive domain calculation in out-of-hospital cardiac arrest (OHCA) survivors (*n* = 108) and myocardial infarction (MI) controls (*n* = 92).**

	M (*SD*)	Range		Percentile						
		Min	*Max*	2	5	25	50	75	95	98
**WAIS-IV Vocabulary**										
OHCA, raw score (*Min–Max* = 0–57)	34.28 (10.67)	11	57	16	17	27	34	43	51	53
OHCA, scaled score (*M* = 10, *SD* =3)	9.58 (3.06)	4	19	5	5	7	9	11	15	16
MI, raw score (*Min–Max* = 0–57)	37.40 (8.83)	18	56	22	23	32	38	43	52	54
MI, scaled score (*M* = 10, *SD* = 3)	10.46 (2.73)	5	19	6	7	9	10	12	15	17
**D-KEFS Verbal Fluency, Letter Fluency**										
OHCA, raw score (*Min–Max* = 0–∞)	37.89 (14.27)	4	83	13	16	29	39	46	60	74
OHCA, scaled score (*M* = 10, *SD* = 3)	10.42 (4.01)	1	19	3	4	8	11	13	17	19
MI, raw score (*Min–Max* = 0–∞)	38.50 (14.70)	6	91	11	14	28	38	47	61	67
MI, scaled score (*M* = 10, *SD* = 3)	10.68 (4.22)	1	19	3	4	8	11	14	17.5	19
**D-KEFS Verbal Fluency, Category Fluency**										
OHCA, raw score (*Min–Max* = 0–∞)	38.92 (11.28)	12	63	18	22	31	39	46	60	61
OHCA, scaled score (*M* = 10, *SD* = 3)	11.19 (4.30)	1	19	2	5	8	11	14	19	19
MI, raw score (*Min–Max* = 0–∞)	41.64 (9.72)	19	65	21	27	36	41	49	59	59
MI, scaled score (*M* = 10, *SD* = 3)	12.29 (3.76)	2	19	5	6	10	12	14	18	19
**WAIS-IV Block Design**										
OHCA, raw score (*Min–Max* = 0–66)	38.13 (12.68)	6	65	16	20	28	37	47	59	61
OHCA, scaled score (*M* = 10, *SD* = 3)	10.42 (2.89)	2	18	5	6	8	11	12	15	16
MI, raw score (*Min–Max* = 0–66)	39.16 (10.81)	6	62	23	24	32	39	47	56	59
MI, scaled score (*M* = 10, *SD* = 3)	10.72 (2.59)	3	17	6	7	9	11	12	15	16
**WAIS-IV Matrix Reasoning**										
OHCA, raw score (*Min–Max* = 0–26)	14.56 (4.67)	5	24	6	8	11	15	18	22	23
OHCA, scaled score (*M* = 10, *SD* = 3)	9.64 (2.85)	3	16	4	5	8	9	11	15	16

MI, raw score (*Min–Max* = 0–26)<br>MI, scaled score (*M* = 10, *SD* = 3)	16.2 (4.45)<br>10.62 (2.79)	6<br>4	23<br>16	7<br>4	8<br>6	14<br>9	17<br>11	19<br>13	22<br>15	22<br>15
**WAIS-IV Digit Span**										
OHCA, raw score (*Min–Max* = 0–48)<br>OHCA, scaled score (*M* = 10, *SD* = 3)	24.49 (5.36)<br>9.68 (2.77)	14<br>4	41<br>18	15<br>5	17<br>6	21<br>8	24<br>9	27<br>11	34<br>15	38<br>17
MI, raw score (*Min–Max* = 0–48)<br>MI, scaled score (*M* = 10, *SD* = 3)	24.27 (4.95)<br>9.65 (2.42)	9<br>5	42<br>19	15<br>6	17<br>6	21<br>8	24<br>10	27<br>11	31<br>14	34<br>16
**WMS-III Spatial Span**										
OHCA, raw score (*Min–Max* = 0–32)<br>OHCA, scaled score (*M* = 10, *SD* = 3)	14.31 (3.94)<br>10.31 (3.54)	5<br>3	24<br>18	7<br>3	8<br>5	12<br>7	14<br>11	17<br>13	22<br>16	23<br>17
MI, raw score (*Min–Max* = 0–32)<br>MI, scaled score (*M* = 10, *SD* = 3)	14.53 (3.07)<br>10.72 (2.97)	8<br>4	23<br>17	9<br>5	10<br>6	12<br>8	14<br>11	16<br>13	20<br>15	21<br>16
**RAVLT Total Recall**										
OHCA, raw score (*Min–Max* = 0–75)<br>OHCA, *z*–score (*M* = 0, *SD* = 1)	40.29 (11.22)<br>-0.39 (1.31)	16<br>-3.40	64<br>2.79	20<br>-3.21	21<br>-2.64	33<br>-1.21	40<br>-0.41	48<br>0.60	59<br>1.49	62<br>1.89
MI, raw score (*Min–Max* = 0–75)<br>MI, *z*-score (*M* = 0, *SD* = 1)	42.68 (9.49)<br>-0.08 (1.04)	17<br>-2.60	69<br>2.50	23<br>-2.34	26<br>-1.81	37<br>-0.86	43<br>-0.05	50<br>0.79	57<br>1.85	58<br>2.28
**RAVLT Delayed Recall**										
OHCA, raw score (*Min–Max* = 0–15)<br>OHCA, *z*-score (*M* = 0, *SD* = 1)	7.67 (3.40)<br>-0.34 (1.18)	0<br>-3.09	15<br>3.33	1<br>-2.48	2.2<br>-2.2	5<br>-1.25	7<br>-0.42	10<br>0.42	13<br>1.55	15<br>1.71
MI, raw score (*Min–Max* = 0–15)<br>MI, *z*-score (*M* = 0, *SD* = 1)	8.4 (3.13)<br>-0.08 (1.04)	1<br>-2.6	15<br>2.5	2<br>-1.95	4<br>-1.63	6<br>-0.83	8<br>-0.27	10<br>0.73	14<br>1.67	15<br>2.07
**WMS-III Logical Memory I**										
OHCA, raw score (*Min–Max* = 0–75)<br>OHCA, scaled score (*M* = 10, *SD* = 3)	33.25 (10.43)<br>9.04 (3.38)	7<br>1	56<br>15	11<br>2	16<br>3	26<br>7	34<br>9	41<br>12	50<br>14	52<br>15
MI, raw score (*Min–Max* = 0–75)<br>MI, scaled score (*M* = 10, *SD* = 3)	35.13 (9.84)<br>9.71 (3.09)	9<br>3	58<br>16	16<br>3	19<br>5	29<br>8	36<br>10	42<br>12	51<br>15	52<br>15

**WMS-III Logical Memory II**										
OHCA, raw score (*Min–Max* = 0–50)	19.55 (7.89)	1	36	3	7	14	19	25	33	35
OHCA, scaled score (*M* = 10, *SD* = 3)	10.02 (3.05)	2	16	3	5	8	10	12	15	15
MI, raw score (*Min–Max* = 0–75)	21.09 (7.80)	2	41	8	10	16	22	26	34	36
MI, scaled score (*M* = 10, *SD* = 3)	10.72 (3.04)	4	18	4.82	6	9	11	13	16	16.2
**BVMT-R Total Recall**										
OHCA, raw score (*Min–Max* = 0–36)	17.29 (7.47)	1	34	3	4	12	17	23	30	30
OHCA, *T*-score (*M* = 50, *SD* = 10)	40.82 (13.74)	10	73	10	10	34	41	51	64	68
MI, raw score (*Min–Max* = 0–36)	19.64 (6.20)	4	32	9	12	15	19	25	30	32
MI, *T*-score (*M* = 50, *SD* = 10)	45.45 (10.70)	21	68	26	28	37	44	54	64	67
**BVMT-R Delayed Recall**										
OHCA, raw score (*Min–Max* = 0–12)	7.30 (3.14)	0	12	0	2	5	7	10	12	12
OHCA, *T*-score (*M* = 50, *SD* = 10)	44.94 (14.04)	10	67	10	22	36	46	58	64	66
MI, raw score (*Min–Max* = 0–12)	8.35 (2.80)	2	12	3	3	7	8	11	12	12
MI, *T*-score (*M* = 50, *SD* = 10)	50.21 (11.73)	23	68	25	30	43	50	61	67	67
**TMT A**										
OHCA, raw score (*Min–Max* = ∞–0)	37.19 (20.67)	145	11	109	75	42	33	25	18	16
OHCA, *z*-score (*M* = 0, *SD* = 1)	-0.07 (1.88)	-11.7	2.09	-5.57	-2.12	-0.56	0.33	1.03	1.53	1.86
MI, raw score (*Min–Max* = ∞–0)	34.72 (15.40)	148	15	58	53	39	32	26	21	21
MI, *z*-score (*M* = 0, *SD* = 1)	0.13 (1.03)	-5.57	2.34	-2.04	-1.50	-0.39	0.36	0.83	1.30	1.34
**D-KEFS Color Word Interference Test, Color Naming**										
OHCA, raw score (*Min–Max* = 90–0)	34.32 (7.51)	59	20	52	49	38	33	28	25	24
OHCA, scaled score (*M* = 10, *SD* = 3)	8.95 (3.02)	1	15	1	3	7	9	11	13	14
MI, raw score (*Min–Max* = 90–0)	33.40 (6.41)	52	19	48	46	37	32	29	26	24
MI, scaled score (*M* = 10, *SD* = 3)	9.32 (2.83)	1	16	2.56	5	8	10	11	13	14
**D-KEFS Color Word Interference Test, Word Reading**										

OHCA, raw score (*Min–Max* = 90–0)	25.36 (5.61)	52	12	38	35	28	25	22	19	16
OHCA, scaled score (*M* = 10, *SD* = 3)	9.25 (2.77)	1	15	2	4	8	10	11	13	14
MI, raw score (*Min–Max* = 90–0)	25.3 (4.58)	38	16	36	34	28	25	22	19	18
MI, scaled score (*M* = 10, *SD* = 3)	9.32 (2.53)	3	15	4	4	7	10	11	13	14
**TMT B**										
OHCA, raw score (*Min–Max* = ∞–0)	104.04 (85.36)	710	29	312	260	110	79	61	40	37
OHCA, *z*-score (*M* = 0, *SD* = 1)	-1.03 (3.83)	-30.51	2.91	-9.29	-5.05	-1.78	-0.13	0.89	1.56	1.76
MI, raw score (*Min–Max* = ∞–0)	86.06 (38.88)	304	40	174	151	94	77	66	45	42
MI, *z*-score (*M* = 0, *SD* = 1)	-0.39 (1.74)	-7.51	1.84	-5.78	-3.58	-1.20	-0.13	0.85	1.47	1.60
**D-KEFS Color Word Interference Test, Inhibition**										
OHCA, raw score (*Min–Max* = 180–0)	68.73 (24.75)	177	25	150	118	76	62	53	45	43
OHCA, scaled score (*M* = 10, *SD* = 3)	9.67 (3.34)	1	14	1	3	9	11	12	14	14
MI, raw score (*Min–Max* = 180–0)	67.33 (23.05)	180	26	128	112	76	61	53	42	40
MI, scaled score (*M* = 10, *SD* = 3)	9.81 (3.58)	1	17	1	2	8	11	12	14	15
**D-KEFS Color Word Interference Test, Inhibition Total Errors**										
OHCA, raw score (*Min–Max* = ∞–0)	1.73 (3.54)	20	0	15	8	2	0	0	0	0
OHCA, scaled score (*M* = 10, *SD* = 3)	10.62 (2.85)	1	13	1	4	10	12	12	13	13
MI, raw score (*Min–Max* = ∞–0)	1.06 (2.05)	16	0	4	4	2	0	0	0	0
MI, scaled score (*M* = 10, *SD* = 3)	11.17 (2.00)	1	13	6	8	10	12	12	13	13

WAIS-IV, Wechsler Adult Intelligence Scale – Fourth Edition; D-KEFS, Delis-Kaplan Executive Function System; WMS-III, Wechsler Memory Scale – Third Edition; RAVLT, Rey Auditory Verbal Learning Test; BVMT-R, Brief Visuospatial Memory Test-Revised; TMT, Trail Making Test.

Notes: Missing were few (≤4) on all *z*-scores. Raw scores are unadjusted while scaled scores, *z*-scores, and *T*-scores are adjusted for age (as well as education for the TMT). On the BVMT-R Total recall and Delayed recall, *T*-scores with the lowest value in the manual, <20, have been transformed to 10 to enable standardized analyses. Numeric low raw scores represent better performance on the TMT and D-KEFS Color Word Interference Test; the *Min–Max* and percentile values in the table reflect this to facilitate comparison between raw and standardized scores.

Supplementary Table 2. **Exploratory analyses on demographic and medical background variables for included out-of-hospital cardiac arrest (OHCA) survivors with major cognitive impairment (z -≤2) in at least one cognitive domain (n = 15) and included OHCA survivors (n = 93) without major cognitive impairment.**

	Included OHCA survivors with major cognitive impairment	Included OHCA survivors without major cognitive impairment	U	p
**Sociodemographic data**				
Age in years at time of examination, median (Q_1–Q_3)	59 (56–68)	63 (57–71)	778	0.50
Male, *n* (%)	13 (87)	82 (88)		1.00
University-level education, with or without degree, *n* (%)	5 (33)	37 (40)		0.80
Previous neurological disease, *n* (%)	3 (21)	5 (5)		0.07
**Prehospital variables**				
Bystander-performed cardiopulmonary resuscitation, *n* (%)	10 (67)	84 (90)		0.02*
First monitored rhythm shockable, *n* (%)	13 (87)	83 (89)		0.70
Time in minutes from arrest to sustained ROSC, median (Q_1–Q_3)	25 (20–42)	20 (13–30)	526	0.10
**In-hospital and rehabilitation data**				
Days at hospital, median (Q_1–Q_3)	33 (24–41)	14 (11–26)	258	<0.001***
Days at intensive care unit, median (Q_1–Q_3)	6 (5–12)	3 (2–6)	328	0.001**
Participation in rehabilitation interventions after cardiac event				
Cardiac rehabilitation, *n* (%)	1 (7)	23 (25)		0.20
Neurorehabilitation, *n* (%)	5 (33)	15 (16)		0.10
Other, *n* (%)	5 (33)	41 (38)		0.60
**At time of examination**				
Hypertension, *n* (%)	9 (60)	60 (65)		0.80
Diabetes, *n* (%)	4 (27)	9 (10)		0.08
Pharmaceutical treatment				
Agitation/anxiety, *n* (%)	4 (27)	4 (4)		0.01*

Depression, *n* (%)	5 (33)	5 (5)		0.004**
Insomnia, *n* (%)	5 (33)	9 (10)		0.02*
HADS Anxiety Subscale ≥8, *n* (%)	6 (40)	16 (17)		0.08
HADS Depression Subscale ≥8, *n* (%)	6 (40)	10 (11)		0.01*
MFI-20 General Fatigue Subscale, median (Q_1–Q_3)	11 (10–17)	10 (7–13)	463	0.09
MFI-20 Physical Fatigue Subscale, median (Q_1–Q_3)	14 (11–16)	11 (6–14)	385	0.02*
MFI-20 Reduced Activity Subscale, median (Q_1–Q_3)	13 (12–18)	9 (6–12)	272	<0.001***
MFI-20 Reduced Motivation Subscale, median (Q_1–Q_3)	12 (9–15)	7 (5–10)	312	0.002**
MFI-20 Mental Fatigue Subscale, median (Q_1–Q_3)	12 (10–14)	7 (4–9)	304	0.001**
MISS ≥6, *n* (%)	4 (27)	20 (21)		0.70

* indicates statistical significance $p < 0.05$

** indicates statistical significance $p < 0.01$

*** indicates statistical significance $p < 0.001$

Q_1–Q_3, quartile 1 to quartile 3; ROSC, return of spontaneous circulation; MoCA, Montreal Cognitive Assessment; HADS, Hospital Anxiety and Depression Scale; MFI-20, Multidimensional Fatigue Inventory; MISS, Minimal Insomnia Symptom Scale.

Note: In variables with no performed Wilcoxon Mann-Whitney *U* test, these were analyzed with Fisher's exact test instead.

***Supplementary Table 3.* Correlation matrix on neuropsychological composite scores and emotional problems, fatigue, and cardiovascular risk factors for out-of-hospital cardiac arrest (OHCA) survivors (*n* = 108) and myocardial infarction (MI) controls (*n* = 92), calculated with Spearman's rho.**

	Verbal composite score	Visual/constructive composite score	Working memory composite score	Episodic memory composite score	Processing speed composite score	Executive functions composite score
**HADS Anxiety Subscale**						
OHCA	-0.04 (0.66)	-0.15 (0.07)	-0.07 (0.42)	-0.01 (0.89)	-0.10 (0.32)	-0.21 (0.01**)
MI	-0.15 (0.19)	-0.16 (0.08)	-0.03 (0.67)	-0.04 (0.73)	-0.17 (0.09)	-0.27 (0.01**)
**HADS Depression Subscale**						
OHCA	-0.15 (0.11)	-0.14 (0.11)	-0.13 (0.20)	-0.13 (0.11)	-0.27 (0.01**)	-0.37 (<0.001***)
MI	-0.32 (<0.001***)	-0.06 (0.46)	0.06 (0.65)	-0.17 (0.10)	-0.22 (0.01**)	-0.26 (0.01**)
**MFI-20 General Fatigue Subscale**						
OHCA	-0.07 (0.42)	-0.04 (0.70)	0.03 (0.78)	0.00 (0.88)	-0.17 (0.09)	-0.24 (0.01**)
MI	-0.28 (<0.001***)	-0.19 (0.05)	-0.02 (0.81)	-0.08 (0.49)	-0.16 (0.09)	-0.18 (0.01**)
**MFI-20 Physical Fatigue Subscale**						
OHCA	-0.16 (0.04)	-0.05 (0.64)	-0.10 (0.20)	-0.08 (0.40)	-0.26 (0.01**)	-0.24 (0.01**)
MI	-0.25 (0.01**)	-0.14 (0.13)	-0.02 (0.81)	-0.08 (0.43)	-0.02 (0.79)	-0.10 (0.22)
**MFI-20 Reduced Activity Subscale**						
OHCA	-0.15 (0.07)	-0.18 (0.05)	-0.20 (0.01**)	-0.20 (0.03*)	-0.31 (<0.001***)	-0.32 (<0.001***)
MI	-0.18 (0.08)	-0.09 (0.39)	0.01 (0.95)	-0.08 (0.48)	-0.03 (0.76)	-0.11 (0.14)

**MFI-20 Reduced Motivation Subscale**						
OHCA	-0.17 (0.05)	-0.27 (<0.001***)	-0.19 (0.01**)	-0.12 (0.12)	-0.32 (<0.001***)	-0.38 (<0.001***)
MI	-0.18 (0.12)	-0.06 (0.57)	-0.04 (0.64)	-0.08 (0.47)	-0.07 (0.59)	-0.16 (0.14)
**MFI-20 Mental Fatigue Subscale**						
OHCA	-0.12 (0.17)	-0.16 (0.06)	-0.12 (0.14)	-0.21 (0.03*)	-0.15 (0.17)	-0.25 (0.01**)
MI	-0.26 (0.02*)	-0.12 (0.26)	0.01 (0.92)	-0.09 (0.40)	-0.09 (0.38)	-0.19 (0.06)
**MISS**						
OHCA	0.02 (0.84)	-0.02 (0.82)	0.04 (0.81)	0.08 (0.45)	-0.05 (0.45)	0.07 (0.60)
MI	-0.18 (0.02*)	-0.09 (0.34)	0.13 (0.24)	0.06 (0.42)	-0.12 (0.30)	0.03 (0.94)
**Hypertension**						
OHCA	0.10 (0.30)	-0.12 (0.15)	0.05 (0.64)	-0.04 (0.49)	0.00 (0.93)	0.07 (0.53)
MI	0.02 (0.69)	0.07 (0.50)	0.14 (0.12)	0.22 (0.03*)	0.02 (0.82)	0.10 (0.30)
**Diabetes**						
OHCA	-0.15 (0.07)	-0.23 (0.01**)	-0.18 (0.03*)	-0.13 (0.14)	-0.10 (0.26)	-0.22 (0.02*)
MI	-0.12 (0.36)	-0.21 (0.04*)	-0.15 (0.14)	-0.09 (0.46)	-0.29 (0.02*)	-0.24 (0.03*)

* indicates statistical significance p <0.05

** indicates statistical significance p <0.01

*** indicates statistical significance p <0.001

HADS, Hospital Anxiety and Depression Scale; MFI-20, Multidimensional Fatigue Inventory; MISS, Minimal Insomnia Symptom Scale.

***Supplementary Table 4.* Spearman associations with confidence intervals (CI) for continuous neuropsychological composite scores, and modified Rankin Scale (mRS) scores for out-of-hospital cardiac arrest survivors (*n* = 108).**

	mRS	95% CI		*p*
		LL	UL	
Verbal composite score	-0.35	-0.51	-0.16	<0.001***
Visual/constructive composite score	-0.23	-0.41	-0.04	0.02*
Working memory composite score	-0.02	-0.22	0.17	0.82
Episodic memory composite score	-0.29	-0.48	-0.09	0.01**
Processing speed composite score	-0.08	-0.27	0.14	0.50
Executive functions composite score	-0.23	-0.40	-0.04	0.02*

LL, lower limit; UL, upper limit

* indicates statistical significance p <0.05

** indicates statistical significance p <0.01

*** indicates statistical significance p <0.001

Notes: Distribution of mRS scores, *n* (%): 0 = 35 (32); 1 = 29 (27); 2 = 34 (31); 3 = 6 (6); 4 = 3 (3); 5 = 1 (1). Missing data were few (≤4) on all neuropsychological composite scores. Numeric low mRS scores represent better functional outcome while numeric low neuropsychological composite scores represent worse performance in the respective cognitive domains.
